# Network-Forming Nanoclusters in Binary As–S/Se Glasses: From Ab Initio Quantum Chemical Modeling to Experimental Evidences

**DOI:** 10.1186/s11671-016-1788-8

**Published:** 2017-01-17

**Authors:** M. Hyla

**Affiliations:** 1 Institute of Physics of Jan Dlugosz University of Czestochowa, Al. Armii Krajowej 13/15, 42-200 Czestochowa, Poland; 2 Lviv Scientific-Research Institute of Materials of Scientific Research Company “Carat”, Stryjska str. 202, Lviv, 79031 Ukraine

**Keywords:** Chalcogenide glasses, Nanocluster, Reversibility window, Ab initio calculation

## Abstract

Network-forming As_2_(S/Se)_m_ nanoclusters are employed to recognize expected variations in a vicinity of some remarkable compositions in binary As–Se/S glassy systems accepted as signatures of optimally constrained intermediate topological phases in earlier temperature-modulated differential scanning calorimetry experiments. The ab initio quantum chemical calculations performed using the cation-interlinking network cluster approach show similar oscillating character in tendency to local chemical decomposition but obvious step-like behavior in preference to global phase separation on boundary chemical compounds (pure chalcogen and stoichiometric arsenic chalcogenides). The onsets of stability are defined for chalcogen-rich glasses, these being connected with As_2_Se_5_ (*Z* = 2.29) and As_2_S_6_ (*Z* = 2.25) nanoclusters for As–Se and As–S glasses, respectively. The physical aging effects result preferentially from global phase separation in As–S glass system due to high localization of covalent bonding and local demixing on neighboring As_2_Se_m+1_ and As_2_Se_m−1_ nanoclusters in As–Se system. These nanoclusters well explain the lower limits of reversibility windows in temperature-modulated differential scanning calorimetry, but they cannot be accepted as signatures of topological phase transitions in respect to the rigidity theory.

## Background

Chalcogenide glasses (ChG) of binary As–Ch system (Ch = S, Se) are representatives of disordered covalent network solids, which clearly demonstrate glass-forming tendencies predicted in terminology of *rigidity theory* initially developed by Phillips and Thorpe [1, 2]. Within this approach, the strongest glass-forming ability is a character for ChG possessing structural network with the *number of degrees of freedom* equals to the *number of Lagrangian constraints per atom n*_*c*_ associated with nearest-neighbor bond-bending and stretching forces (so in this case, the short-range configuration entropy and network strain energy tend to zero). In such a way, an *average coordination number of glass network* should be close to *Z* = 2.40 for best glass-forming compounds (i.e., stoichiometric As_2_S_3_ and As_2_Se_3_), which are optimally constrained (*n*_*c*_ = 3) and thus, not affected by physical aging. The optimally constrained (rigid but unstressed) *intermediate phase* (IPh) in ChG is expected in narrow compositional domain between under-constrained floppy phase (FPh, *n*_*c*_ < 3) and over-constrained stressed rigid phase (SRPh, *n*_*c*_ > 3) [3, 4]. In respect to theoretical calculations [4], the typical width of such IPh is expected to be rather narrow in ChG, since the saturated covalent-bonded glassy network exists at a cost of very low entropy as topologically self-organized phase (*n*_*c*_ = 3).

Since the earliest 2000s, Boolchand et al. [5–8] tried to prove experimentally the IPh employing the method of temperature-modulated differential scanning calorimetry (TM-DSC) as probe for ChG with nearly vanishing non-reversing enthalpy (Δ*H*_nr_) forming the so-called *reversibility window* (RW). Nevertheless, the experimentally detectable RW in ChG of binary As–S (*Z* = 2.225 ÷ 2.29) [6] and As–Se (*Z* = 2.29 ÷ 2.37) [6–8] systems occurs essentially shifted towards Ch side and too compositionally stretched to be accepted as realistic IPh signatures. Additionally, the compositional boundaries for RW in TM-DSC experiments were instable, showing an obvious trend to physical aging with prolonged duration [8–12] and changed storage conditions [8, 13, 14]. Furthermore, the notable dependence of aging time scales on the distance from glass transition region [15], which plays a decisive role in view of the known Williams–Landel–Ferry relation [16], was also ignored in these measurements.

Despite this argumentation, testifying that compositional boundaries of IPh in As–S/Se ChG determined as TM-DSC-probed RW [5–8] are rather artifacts of measuring procedure (revealing essential variation in sensitivities to different atomic entities [15, 17, 18]), origin of these compositional anomalies in a vicinity of *Z* = 2.225 (As–S system) and *Z* = 2.29 (As–Se system) remains still controversial. In this work, this specificity for As–S/Se ChG systems will be traced using ab initio quantum chemical modeling known as cation-interlinking network cluster approach (CINCA) [19–22] applied to As_2_(S/Se)_m_ nanoclusters.

Although As–S and As–Se are isotypical ChG systems, their network-forming tendencies differ essentially. Thus, the region of glass formation stretches from *Z* ≅ 2.00 (elemental Se) to *Z* ≅ 2.60 (As_3_Se_2_ glassy alloy) in As–Se system [23–26], whereas it is distinctly narrower in As–S system being in the range of ~2.05 < *Z* < (2.44–2.46) [23–26]. Myers and Felty [26] explained this by different melting behaviors of these systems. The region of stable homogeneous glasses depends on melt-quenching glass preparation technological route. For example, Hruby [27] pointed out that a second glass-forming region in As–S system exists at *Z* = 2.51 ÷ 2.66, when the melt was held for several hours at 300 ÷ 400 °C above the liquidus temperature.

Taking into account the phase diagrams of As–Ch systems (Ch = Se, S) [26, 28, 29], two intrinsic decomposition processes are to be expected for Ch-rich glass compositions (*Z* ≤ 2.40). The first process can be attributed to instability in a glassy network composed by As_2_Ch_m_ atomic clusters interlinked by Ch chains due to local demixing on compositionally close As_2_Ch_m+1_ and As_2_Ch_m−1_ clusters [12], this *local chemical decomposition* obeying scheme:

1 $$ 2\cdotp A{s}_2C{h}_m\leftrightarrow A{s}_2C{h}_{m+1} + A{s}_2C{h}_{m-1} $$

The second decomposition process is connected with global possibility of glassy network to be separated on two distinct phases, the stoichiometric As_2_Ch_3_ and “pure” chalcogen Ch. Noteworthy, this *global phase separation* in As_2_Ch_m_ ChG results in two corner-shared AsCh_3/2_ pyramids (i.e., As_2_Ch_3_ cluster) and Ch_m−3_ remainder according to the reaction [12]:

2 $$ A{s}_2C{h}_m\leftrightarrow A{s}_2C{h}_3 + C{h}_{m-3}. $$

The main goal of this paper is to make comparison and discuss *boundaries of structural phase stability* in As–S/Se ChG in the light of currently available experimental evidences.

## Methods

### Nanoclusters Modeling Procedure

Within most generalized approach, atomic clusters of As_2_(S/Se)_m_ chemical compositions which represented themselves as trigonal As(S/Se)_3/2_ pyramids linked by (S/Se)_m–3_ chains are principal network-forming clusters (NFC) in Ch-rich ChG of As–(S/Se) systems [25, 26, 30]. This simplification allows usage a simple simulation route for relatively small atomic entities and available software like Hyper Chem Release 7.5 program [31, 32], instead of complicated and time-consuming modeling procedures for multi-atomic glassy networks evolved hundreds or even thousands of atoms. The detailed information on main principles of developed modeling route (CINCA) you can find elsewhere [19–22].

This approach applied recently to As–Se ChG based on As_2_Se_m_ NFC [30] shows *instability onset* near *Z* = 2.25, where these glasses demonstrate strong tendency towards both global phase separation due to reaction (2) and local chemical decomposition due to reaction (1). In this work, the same CINCA simulation procedure will be applied to As_2_S_m_ NFC (*m* = 3 ÷ 9), which serves as a basis for S-rich ChG of binary As–S system.

Thus, the glass-forming tendencies in ChG of As–Ch system (Ch = S, Se) will be examined through a row of NFC built so to reflect most essential chemical interactions between high-coordinated cation-like atoms as *branch points* (threefold coordinated As atoms) [22]. Each As atom is supposed to form *cation-centered polyhedron* such as trigonal AsCh_3/2_ pyramid, which (in respect to *chain-crossing* model [25, 26]) is linked with other such pyramid by twofold coordinated Ch atoms. To quantify inter-cluster interaction, the outer part of atomic cluster (*shell*) should be distinguished from the remainder (*core*), and only “pure” interaction between these neighboring cations surrounded by the same cluster shells will be considered. It means that principle of the same *topological shielding* will be employed to reflect network-forming tendencies (the simplest *cluster shell* coincides with boundary of cation-centered cluster itself). Further, we allow slight deviations which enlarge cluster shell by equal amount of additional Ch atoms and reduce the number of Ch atoms between neighboring As cations, until this procedure is possible for chosen ChG composition. Difference in length of cluster core and shell determined by number of Ch half-atoms Δ*l* describes deviation of network-forming tendency from chain-crossing model (Δ*l* = 0 corresponds to chain-crossing arrangement, when all As atoms in cores and shells are interconnected by equivalent Ch_n_ chains). In this research, we consider only integer Δ*l* values proper to symmetric clusters (with equivalent legs in cluster shell), while fractional ones proper to asymmetric clusters with different legs in shell are ignored. We suppose these asymmetric clusters will be ranged in their cluster-forming energy (CFE) between corresponding values for neighboring symmetric clusters. This simplification allows reliable quantum chemical calculations for smaller symmetric clusters with equivalent inter-cluster legs in cluster shells.

Thus, the CFE, i.e., energy of geometrically optimized NFC averaged per atom, is probed using the CINCA modeling supported by ab initio RHF/6-311G* calculations (this basis set is chosen as reliable for all clusters, despite their length and symmetry) [19–22]. The geometrical optimization and single-point energy calculations are carried out with the Fletcher–Reeves conjugate gradient method until root-mean-square gradient of 0.1 kcal/Å mol is reached (the CFE is also corrected on H atoms terminated outer Ch atoms).

## Results and Discussion

The average CFE for As_2_S_m_ NFC (*m* = 3 ÷ 9) in As–S system are gathered in Table 1 along with previously calculated data for As_2_Se_m_ NFC (*m* = 3 ÷ 7) in As–Se ChG system [30]. Numerical values of CFE for geometrically optimized AsSe_3/2_ (*E*_*f*_ = −72.309 kcal/mol) and AsS_3/2_ (*E*_*f*_ = −79.404 kcal/mol) pyramids were taken as reference points for respective ChG.

**Table 1:** Average CFE for geometrically optimized As_2_S_m_ NFC in As–S system as compared with analogous data for As_2_Se_m_ NFC in As–Se ChG [30] (best CFE are italicized)

Nanocluster	*Z*	*E*_*f*_ , kcal/mol						
		Δ*l* = 0	Δ*l* = 1	Δ*l* = 2	Δ*l* = 3	Δ*l* = 4	Δ*l* = 5	Δ*l* = 6
As–Se system								
As_2_Se_3_	2.40	*0.31*						
As_2_Se_4_	2.33		*−0.76*					
As_2_Se_5_	2.29		−1.05	*−0.95*				
As_2_Se_6_	2.25	−2.47			*−1.96*			
As_2_Se_7_	2.22		*−2.10*	−2.19		−2.33		
As–S system								
As_2_S_3_	2.40	*0.004*						
As_2_S_4_	2.33		*−1.06*					
As_2_S_5_	2.29		*−0.76*	−1.75				
As_2_S_6_	2.25	*−1.43*			−2.25			
As_2_S_7_	2.22		*−1.84*	−3.39		−2.64		
As_2_S_8_	2.20		−3.68	*−2.46*			−3.14	
As_2_S_9_	2.18	−3.57			*−2.30* −2.72			−3.34

**Fig. 1:**
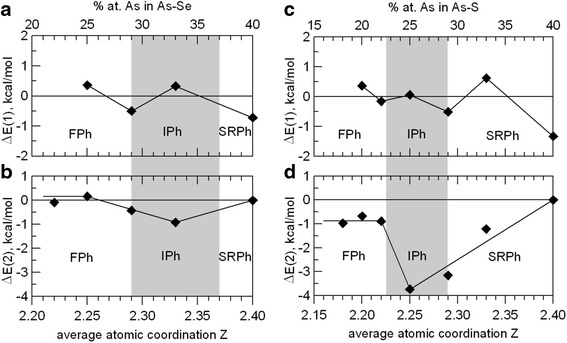
Compositional dependencies of energetic barriers of local chemical decomposition (**a**, **c**) and global phase separation (**b**, **d**) for geometrically optimized NFC in As–Se (**a**, **b**) and As–S (**c**, **d**) glassy systems (compositional domains of expected optimally constrained phase determined in respect to TM-DSC [5–8] are *shadowed*)

The energetic barriers of intrinsic decomposition processes, denoted as Δ*E*(1) or Δ*E*(2) in accord to reactions (1) and (2), were calculated using bold-distinguished values of best CFE from Table 1. In calculations concerning decomposition of stoichiometric As_2_S_3_, the CFE of geometrically optimized As_2_S_4/2_ NFC (*m* = 2) based on bridging homonuclear As–As chemical bonds (*E*_*f*_ = −77.683 kcal/mol) was taken into account. To estimate an energetic preference of global chemical decomposition due to reaction (2), the CFE for geometry-optimized S_m−3_ and Se_m−3_ nanoclusters were taken from [30, 33]. The corresponding compositional dependencies of energetic barriers Δ*E*(1) and Δ*E*(2) for best geometrically optimized NFC in As–Se/S ChG are depicted in Fig. 1 (the positive values are accepted for right-shifted equilibria, and errors are within symbol points).

It is seen that As_2_Ch_m_ nanoclusters in both ChG systems show similar oscillating behavior in tendency to local chemical decomposition in respect to reaction (1). This decomposition on As_2_Ch_m+1_ and As_2_Ch_m−1_ clusters is energetically favorable for ChG with *Z* = 2.33, *Z* = 2.25, and *Z* = 2.20, while ChG of stoichiometric As_2_Ch_3_ (*Z* = 2.40) and As_2_Ch_5_ (*Z* = 2.29) compositions are stable against decomposition showing notable negative values of Δ*E*(1) barriers (Fig. 1a, c).

As to global phase separation on corner-shared AsCh_3/2_ pyramids (i.e., NFC of As_2_Ch_3_ composition) and Ch_m−3_ remainder (i.e., NFC of “pure” Ch_2/2_ chemical composition) in respect to reaction (2), the behavior of examined As–Se and As–S ChG is principally different.

With going from Ch-rich compositions towards stoichiometry (i.e., As_2_Ch_3_ composition) in As–Se system, the most compliant ChG correspond to lowest *Z* (2.22 and 2.25), while other glasses with *Z* = 2.29, 2.33, and 2.40 are rather resistant to decomposition. This tendency is very weak because of the small difference in Δ*E*(2) barriers not exceeding 1.1 kcal/mol, but it is quite stable, especially as compared in a sum of two Δ*E*(1) and Δ*E*(2) barriers. In final, we can distinguish the As_2_Se_5_ glass (*Z* = 2.29), installing onset to As–Se ChG, which are most resistant against decomposition in respect to both reactions (1) and (2). Noteworthy, this As_2_Se_5_ composition coincides with beginning of RW (i.e., the rigidity transition) in binary As–Se ChG determined in TM-DSC experiments [6–8].

In contrast, decomposition processes are more significant in As–S ChG. Despite energetic barriers of global phase separation Δ*E*(2) in this system attain only negative values, this parameter clearly shows *threshold-like behavior* in a vicinity of *Z* = 2.22–2.25 (Fig. 1d). Initially, at higher Ch content (*Z* = 2.18, 2.20, 2.22), these ChG are rather well subjected to phase separation with nearly the same possibility defined by Δ*E*(2) ≅ −0.9 kcal/mol. But with further tending towards stoichiometry, the As−S ChG becomes resistant to global phase separation in respect to reaction (2), this transition being revealed abruptly just near *Z* = 2.25. The latter seems to be responsible for eventually earlier rigidity transition in this ChG system at *Z* = 2.225 as detected using TM-DSC [6]. So, the glasses of As_2_S_6_ composition (*Z* = 2.25) can be accepted as introducing the compositional onset for decomposition-stable As-S ChG.

**Fig. 2:**
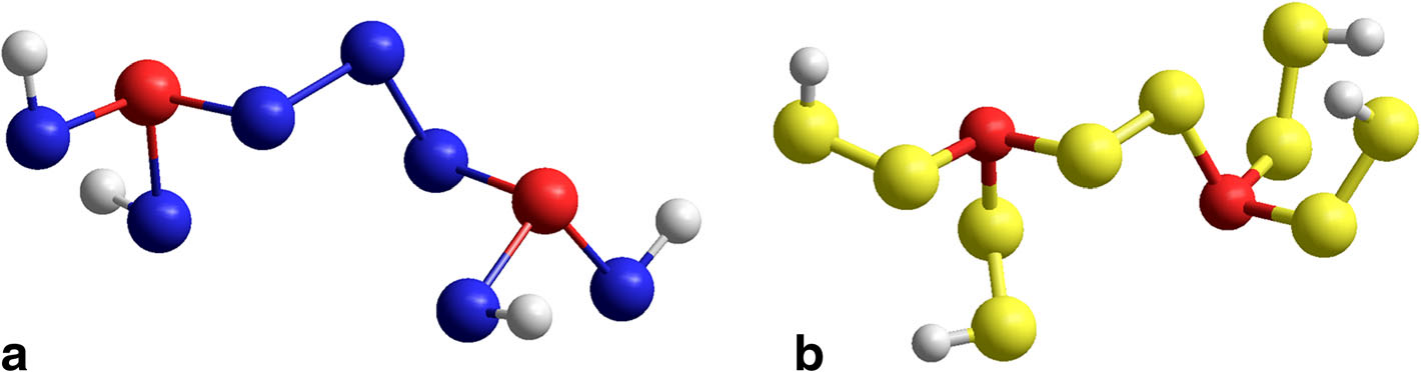
Geometrically optimized configurations of NFC introducing stability onset in As–Se/S ChG: **a** As_2_Se_5_ (*Z* = 2.29, Δ*l* = 2); **b** As_2_S_6_ (*Z* = 2.25, Δ*l* = 0)

The geometrically optimized configurations of these NFC, i.e., As_2_Se_5_ (*Z* = 2.29, Δ*l* = 2) and As_2_S_6_ (*Z* = 2.25, Δ*l* = 0), introducing stability onsets in Ch-rich As-Se/S ChG, are presented in Fig. 2a and b, respectively, and the corresponding structural parameters of these glasses being given in Table 2. The model bond distances and angles for these nanoclusters occur to be close to the ones proper for known experimental prototypes [34, 35].

**Table 2:** Geometrical parameters of optimized NFC introducing stability onset in As-Se/S ChG: As_2_Se_5_ (*Z* = 2.29, Δ*l* = 2) and As_2_S_6_ (*Z* = 2.25, Δ*l* = 0)

Nanocluster, *Z*	Bond distance, [⋅10^−4^ nm]		Bond angle, [deg]		
	As–Ch	Ch–Ch	∠Ch–As–Ch	∠Ch–Ch–As	∠Ch–Ch–Ch
As_2_Se_5_, *Z* = 2.29	2394 2399 2404 2404 2393 2399	2347 2347	97.4 96.1 97.6 96.3 97.5 97.6	99.3 99.1	105.7
Average	2399	2347	97.1	99.2	105.7
As_2_S_6_, *Z* = 2.25	2264 2262 2267 2261 2263 2262	2086	102.9 103.7 93.1 105.9 102.5 100.1	99.6 99.7	–
Average	2263	2086	101.3	99.7	*–*

Specifically, in binary As–Se system, the stability onset corresponds to As_2_Se_5_ (*Z* = 2.29, Δ*l* = 2) NFC, introducing *locally inhomogeneous* structural network, where each AsSe_3/2_ pyramid is connected with neighboring ones through two short =As–Se–As= bridges (i.e., single-atom Ch chain) and one long =As–Se–Se–Se–As= linkages (i.e., triple atoms Ch chain). That is why the As_3_Se_7_ glass corresponding to *Z* = 2.30, which is nominally composed of trigonal AsSe_3/2_ pyramids interlinked via double-atoms Ch chains (i.e., =As–Se–Se–As= units), demonstrates an obvious propensity to long-term physical aging due to local decomposition on short =As–Se–As= and long =As–Se–Se–Se–As= chain-like structural fragments, as it was convincingly evidenced from recent Raman scattering, NMR and high-resolution XPS measurements [36, 37]. More generally, the local chemical decomposition by reaction (1) dominates over global phase separation by reaction (2) in binary As–Se ChG, thus meaning shift in the stability onset of this system towards Ch-less compositions (*Z* ≥ 2.29).

Alternatively, the ChG of binary As–S system demonstrates an obvious tendency to keep ideal chain-crossing arrangement built of As_2_S_6_ NFC (*Z* = 2.25, Δ*l* = 0) character for highly *homogeneous* structural network, where all trigonal AsS_3/2_ pyramids are interconnected via the same double-Ch atoms =As–S–S–As= links. The global phase separation by reaction (2) prevails over the local chemical decomposition by reaction (1) in this system (Fig. 1c, d), thus resulting in S-phase extraction for S-rich compositions with *Z* < 2.22, which is in an excellent harmony with well-known experimental data [23–25, 37–39]. In terms of potential energy landscape [40–42], this binary As–S glass system can be described by distinguished double-well potentials for structural fragments based on double Ch atoms =As–S–S–As= and single Ch atom =As–S–As= linkages. Due to high difference in the energetic barriers that separated these states, this system tends towards Ch-phase extraction at higher S content, but not towards local decomposition on neighboring intrinsic compounds.

Therefore, the effects of long-term physical aging in the studied ChG belonging to the RW defined using TM-DSC [5–8] result preferentially from global phase separation via reaction (1) in As–S system (due to high localization of covalent chemical bonding) and local demixing on compositionally neighboring As_2_Se_m+1_ and As_2_Se_m–1_ clusters via reaction (2) in As–Se system. It means that the IPh determined as TM-DSC-defined RW for both As–Se/S systems [5–8] are only artifacts of this experimental measuring procedure, which have no any relation to realistic topological phase transitions in respect to the rigidity theory [1, 2].

Interestingly, the minimal barriers in global phase separation Δ*E*(2) for both As–S/Se ChG roughly coincide with the center of compositional domains ascribed to the TM-DSC-defined RW [5–8]. Thus, the As_2_Se_4_ (*Z* = 2.33) and As_2_S_6_ (*Z* = 2.29) ChG possess the Δ*E*(2) barriers reaching respectively −0.918 kcal/mol and −3.734 kcal/mol. So it should be expected that long-term physical aging will be also revealed in ChG of these chemical compositions, as it was well documented experimentally [11–13, 18].

The region of stable homogeneous glass-forming ability in ChG is known to be strongly dependent on glass preparation conditions (melting temperature, quenching rate, etc.) [23–26]. The obtained results show energetic preference of some cluster-forming tendencies. The structures of more extended configurations are determined by specifics of covalent chemical bonds with characteristic fluctuations in charge density distribution and bond directionality. The network-forming clusters in this research reflect principal conditions of covalent-like interactions, chemical composition, and space topology for ideal bond-saturated network in thermodynamically equilibrium conditions (there are no surfaces, coordination defects or dangling bonds, etc.). Anomalous preparation conditions provide ChG with different structural defects, which cannot be strictly evaluated within this simplified approach.

## Conclusions

Chalcogen-rich As–S/Se glassy systems are traced using ab initio quantum chemical modeling known as cation-interlinked network cluster approach applied to As_2_(S/Se)_m_ nanoclusters to recognize expected variations in a vicinity of some remarkable compositions accepted as signatures of optimally constrained, rigid but unstressed intermediate topological phases in earlier temperature-modulated differential scanning calorimetry experiments. The examined As_2_(S/Se)_m_ network-forming nanoclusters show similar oscillating character in tendency to local chemical decomposition but obvious step-like behavior in preference to global phase separation on boundary chemical compounds. The global phase separation is dominant in S-rich As–S glasses, keeping chain-crossing arrangement character for highly homogeneous structural networks, while local demixing on compositionally neighboring nanoclusters prevails in Se-rich As–Se glasses possessing inhomogeneous structural networks. The geometrically optimized configurations of As_2_Se_5_ (*Z* = 2.29) and As_2_S_6_ (*Z* = 2.25) nanoclusters introducing onsets of stability in binary As–Se/S systems are simulated. These compositions are shown to coincide well with lower limits of reversibility windows in temperature-modulated differential scanning calorimetry.

## Abbreviations

CFE: Cluster-forming energy
Ch: Chalcogen
ChG: Chalcogenide glasses
CINCA: Cation-interlinking network clusters approach
FPh: Floppy phase
IPh: Intermediate phase
NFC: Network-forming cluster
RW: Reversibility window
SRPh: Stress-rigid phase
TM-DSC: Temperature-modulated differential scanning calorimetry
